# Seed fates in crop–wild hybrid sunflower: crop allele and maternal effects

**DOI:** 10.1111/eva.12236

**Published:** 2014-12-05

**Authors:** Brian A Pace, Helen M Alexander, Jason D Emry, Kristin L Mercer

**Affiliations:** 1 Department of Horticulture and Crop Science, Ohio State UniversityColumbus, OH, USA; 2 Department of Ecology and Evolutionary Biology, University of KansasLawrence, KS, USA; 3 Department of Biology, Washburn UniversityTopeka, KS, USA

**Keywords:** dormancy, gene flow, hybridization, introgression, maternal effects, seed banks, sunflower

## Abstract

Domestication has resulted in selection upon seed traits found in wild populations, yet crop-wild hybrids retain some aspects of both parental phenotypes. Seed fates of germination, dormancy, and mortality can influence the success of crop allele introgression in crop-wild hybrid zones, especially if crop alleles or crop-imparted seed coverings result in out-of-season germination. We performed a seed burial experiment using crop, wild, and diverse hybrid sunflower (*Helianthus annuus*) cross types to test how a cross type's maternal parent and nuclear genetic composition might affect its fate under field conditions. We observed higher maladaptive fall germination in the crop- and F_1_- produced seeds than wild-produced seeds and, due to an interaction with percent crop alleles, fall germination was higher for cross types with more crop-like nuclear genetics. By spring, crop-produced cross types had the highest overwintering mortality, primarily due to higher fall germination. Early spring germination was identical across maternal types, but germination continued for F_1_-produced seeds. In conclusion, the more wild-like the maternal parent or the less proportion of the cross type's genome contributed by the crop, the greater likelihood a seed will remain ungerminated than die. Wild-like dormancy may facilitate introgression through future recruitment from the soil seed bank.

## Introduction

Hybridization is common between crop and wild relatives, with 22 of the 25 most important domesticated plant species hybridizing in some part of their geographical range (Ellstrand 2003; Ellstrand et al. 2013). Gene flow can introduce novel alleles into plant populations and may or may not lead to introgression (the transfer of alleles from one taxon to another; Anderson and Hubricht 1938) depending on the degree to which the novel alleles alter fitness and therefore are selected upon (Slatkin 1987; Lenormand 2002). Neutral processes can also govern introgression (Ellstrand et al. 1999). It has long been assumed that crop alleles should typically reduce crop-wild hybrid fitness relative to wild fitness outside of cultivation (Stewart 2003). For example, domestication traits such as reduced seed shattering would be expected to be disadvantageous in a wild setting. Several empirical studies do show hybrids between crops, and their wild relatives can have reduced fitness relative to their parents (Snow et al. 1998; Hauser and Shim 2007). However, other researchers have demonstrated that alleles from crop populations can improve the fitness of hybrid offspring relative to wilds (Campbell et al. 2006; Sahoo et al. 2010; Hovick et al. 2012) or have no fitness effect (Arriola and Ellstrand 1997). If crop alleles improve the fitness of hybrid progeny, hybrid lineages have the potential to become weedy or invasive (Arriola and Ellstrand 1997; Snow et al. 1998; Ellstrand 2003) or threaten the diversity of important crop genetic resources (Gepts and Papa 2003).

Given that recombination creates new allele combinations after the F_1_ generation, fitness studies should ideally be conducted in the context of the multiple generation nature of the introgression process. While many combinations of wild, hybrid, and crop parents are possible, constituting different cross types, few fitness studies go beyond comparisons of two or three generations (but see Hauser et al. 1998 and Yang et al. 2011). Further, most studies of hybrid fitness have focused on seedling or adult survival or reproduction (Arriola and Ellstrand 1997; Snow et al. 1998; Mercer et al. 2006a). Seed traits of crop-wild hybrids are understudied in both laboratory conditions, and particularly under natural conditions (Linder and Schmitt 1994; respectively and but see Linder 1998). We particularly highlight the importance of studying seed dormancy, defined as a block to germination under otherwise favorable moisture, temperature, and light conditions (Baskin and Baskin 2001). Seed dormancy has the potential to enhance plant fitness and long-term population persistence in wild populations by providing escape from unfavorable conditions and insurance against reproductive failure (Baskin and Baskin 2001; Vitalis et al. 2004; Alexander et al. 2009). Yet in cultivated plants, dormancy is selected against, so reduced dormancy may be a domestication trait that could decrease hybrid fitness outside of cultivation (Stewart 2003). Nevertheless, in a crop-wild hybrid zone, recombination after the F_1_ generation could lead to a range of levels of dormancy in crop-wild hybrids with attending effects on fitness (Mercer et al. 2006a). The presence and fitness of the recombining hybrid generations could be tied to their dormancy characteristics, thereby influencing levels of introgression of linked crop alleles. Even low persistence of hybrid seed in the soil seed bank, for example, may extend the effects of an initial hybridization event, allowing for the continued emergence of hybrids (e.g. F_1_ or backcross) in future years (Snow et al. 1998; Mercer et al. 2007). Seed dormancy, of course, must be considered in the context of other seed fates (e.g. germination, mortality). For example, cross types with high germination will have the opportunity to contribute to the standing population and seed production that year, while high mortality would reduce both. Cross types that exhibit higher dormancy will have fewer seedlings emerging in a given season; however, there could also be recruitment from the seed bank (Cummings et al. 2002), allowing hybrids to play a role in sunflower metapopulation dynamics (Alexander et al. 2012).

Various genetic factors have been shown to influence seed dormancy via GA and ABA regulation (Donohue et al. 2005). For species that have physiological dormancy, germination can be limited via the embryo, seed coat, or seed coverings (e.g. pericarp) until certain temperature or moisture conditions are met, unlike other dormancy types, such as physical dormancy, where impermeable seed coats prevent water entry (Baskin and Baskin 2001). Embryos contain the nuclear genome, with equal genetic contributions from the seed and pollen parents. If embryo genetics completely controls dormancy via GA and ABA-mediated mechanisms, seed dormancy could be predicted by the parental genotypes (Koorneef et al. 2002). For example, if seeds from the crop parent lack seed dormancy and those of the wild parent have extensive dormancy, all crop-wild F_1_ cross types might be hypothesized to have intermediate levels, depending on the dominance levels of the alleles involved. However, seed traits, including dormancy, can be disproportionately affected by the maternal parent, that is, experience genetic maternal effects (Byers et al. 1997). These can be due to matrilineal inheritance of cytoplasm, organelles, and chloroplasts, unequal endosperm contribution, and maternal tissues surrounding the seed during and after development (Roach and Wulff 1987).

To explore the effects of crop-wild hybridization on seed dormancy and other seed fates under field conditions, we focus on annual sunflowers (*Helianthus annuus*). This study system has many advantages. First, there is a rich literature on crop-wild hybridization in sunflower (Arias and Rieseberg 1994; Snow et al. 1998; Burke et al. 2002; Reagon and Snow 2006). Second, cultivated sunflower differs from wild types for several characters, including a lack of seed dormancy. Relatedly, there is extensive information on wild seed dormancy and seed banks (e.g. Burnside et al. 1996; Teo-Sherrill 1996; Alexander et al. 2009, 2014). Third, laboratory research has highlighted variation in seed size and effects of seed coverings on germination behavior for crop, F_1_ hybrid, and wild maternal parents (Brunick 2007; Weiss et al. 2013). In particular, we showed that maternal parent and embryo genetics both had important effects on seed germination in the laboratory (Weiss et al. 2013). Finally, crop-wild hybridization has been shown to reduce dormancy in field studies when compared to wild seeds (Snow et al. 1998; Mercer et al. 2006b; Alexander et al. 2014). However, such field studies were carried out with a limited number of cross types and thus do not fully represent the genetic diversity likely present at early stages of crop-wild hybridization. As a consequence, we cannot fully explore how seed fates vary across diverse crop-wild hybrid generations under field conditions and the degree to which the maternal parent or the nuclear genetics composition determine the patterns we see. For example, we have learned that cross types precociously germinating in the fall should create a significant source of mortality for some hybrids (Alexander et al. 2014). Yet we do not understand how the maternal parent of crop-wild hybrid seed may affect overwinter survival and entry into a persistent seed bank. These results could imply that coexisting hybrid cross types may be more or less able to act as likely routes of crop allele introgression.

To address these gaps in the literature, we studied seed dormancy, germination, and mortality patterns of a diverse array of crop-wild hybrids. Our study asks to what degree are maternal effects, the genetics of the embryo, or interactions between these factors, responsible for seed fates in sunflower crop-wild hybrids? To address this question, we generated 15 cross types ranging from pure wild to crop with numerous hybrids in between, all produced on wild, F_1_, or crop parents. Our crossing design allowed us to explore not only nuclear and maternal genetic effects, but also potential interactions by comparing reciprocal cross types and many cross types of each maternal type. We evaluated cross type performance under field conditions using a seed burial study where we removed packets at three time periods to assess (i) fall germination, (ii) early spring germination and overwinter mortality, and (iii) peak germination and dormancy. Given the crucial role of seed traits and seed banks in plant populations, we expect that our results will inform understanding of the potential for crop allele introgression.

## Methods

### Study system

Sunflower (*Helianthus annuus*), a native to North America, was domesticated in a single event (Harter et al. 2004). Wild (or common) sunflower colonizes disturbance, invades agricultural fields (Seiler and Rieseberg 1997; Mesbah et al. 2004), and can form seed banks (Alexander et al. 2009). Overlapping flowering times and shared pollinators allow for gene flow between weedy and wild sunflower, as well as crop varieties (Arias and Rieseberg 1994; Burke et al. 2002), and introgression of crop alleles has been noted (Whitton et al. 1997). Many wild contribute beneficial agronomic traits, making populations of wild sunflower an *in situ* repository for alleles not represented in breeding lines (Seiler 2007).

Sunflower achenes (hereafter seeds) are produced by individual flowers within an inflorescence (hereafter head) and consist of the embryo, the testa, and a protective pericarp, which conforms to the seed surface. The pericarp is maternal tissue that is composed of layers of parenchyma cells (Seiler 1997). Most of the volume of a sunflower embryo is accounted for by partially formed storage cotyledons, with the comparatively small embryo located at the micropylar (or pointed) end.

### Experimental seed source

As described in Weiss et al. (2013), we performed all crossing to produce seeds for this experiment at Waterman Farm at The Ohio State University in Columbus, Ohio in 2010 under uniform conditions with one application of 75-50-100 NPK fertilizer. All paternal and maternal parents for seeds generated in 2010 were derived from a previous round of hand pollinations (2009) as described in Alexander et al. (2014). We generated seeds of 15 sunflower cross types on wild, F_1_ crop-wild hybrid, and crop maternal plants (Table1). Crop parents were from inbred line HA89 (USDA, Fargo, North Dakota), and wild parents descended from 10 populations collected around Lawrence, Kansas within 5–30 km of the experimental site. Several of these cross types were reciprocal cross type pairs of the same maternal and paternal parents, while others had similar nuclear genetic composition with differing maternal parents (Table1). All heads were protected from spontaneous cross-pollination to ensure the integrity of the crosses. Due to the large number of crosses to be performed the size of mature sunflower plants and the number of flowers they can produce, we used two methods to exclude pollinators: either pollinator exclusion bags or mesh-covered cages. As crop plants can self-pollinate, they were also emasculated daily prior to stigma emergence prior to acting as maternal plants.

**Table 1:** Parental cross types were made in 2009 with hand pollinations. In 2010, cross types were produced with hand pollination for use in seed burial experiment. For all cross types, the maternal parent listed first and the paternal parent listed second

Paternal parent	Maternal parent		
	Wild: W × W	F_1_: W × C	Crop: C × C
Wild: W × W	0+ W × W	25% F_1_ × W*	50% C × W*
BC: W × F1 or F1 × W	12.5% W × BC	37.5% F_1_ × BC	62.5% C × BC
F_1_: W × C	25% W × F_1_*	50% F_1_ × F_1_	75% C × F_1_*
F_2_: F_1_ × F_1_	25% W × F_2_	50% F_1_ × F_2_	75% C × F_2_
Crop: C × C	50% W × C*	75% F_1_ × C*	100% C × C

Cross types marked with *are part of reciprocal cross type pairs with the same % crop alleles but different maternal parents used in contrast comparisons.

To sample across the genetic diversity inherent in our materials, we bulked seed for a given cross type from multiple heads from multiple maternal plants. However, there were various limitations for producing each cross type. For cross types produced on wild and F_1_ maternal plants, we bulked equal numbers of seed from two to three heads from eight maternal plants for each cross type. For cross types produced on crop maternal plants, pollen parent diversity was emphasized due to the homogeneity of the HA89 inbred line. Here, we used eight pollen parents for all crop-produced cross types except C × W (Table1), where four were used. Greater than four pollen parents from W × C were possible through the use of pollen frozen in liquid nitrogen and stored at −80°C from earlier in the season.

### Field seed burial experiment

We designed a seed burial study to evaluate seed germination, dormancy, and overwinter seed survival for each cross type under field conditions. We created seed burial strips using 20 × 105 cm strips of no-see-um netting (315 holes/cm^2^) to be removed at three time periods. We folded strips in half and created 15 separate 7 × 10 cm compartments using a high-temperature hot glue gun and randomly assigned the 15 cross types to each compartment. We filled compartments with 20 seeds for cross types produced on wild plants and F_1_ maternal plants, and 15 seeds for cross types produced on crop maternal plants due to seed limitations. A total of 2475 seeds were used.

During the third week of November, 2010, we buried 45 strips at a 10 cm depth of soil in a roto-tilled 5.4 ha brome field at the University of Kansas Field Station (Jefferson County, Kansas). We used a split-plot design with 15 blocks. Within each block, date of removal (applied to a given strip) was the main plot, and cross type (applied to individual seed packets within each strip) was the subplot. The three removal dates were as follows: late fall (removed on December 10, 2010), for monitoring maladaptive fall germination; early spring (removed on March 8, 2011), for investigating early germination; and spring (removed on April 8, 2011), for capturing peak emergence. Removal dates were selected based on a previous seed burial study at this location conducted from 2009 to 2010 (Alexander et al. 2014; Mercer et al. 2014). Each block was then covered by hardware cloth to prevent seed predation.

At each removal date, we evaluated all seeds as a single cohort by block within 48 h at The Ohio State University in Columbus, Ohio. Within each compartment, we noted the number of dead (putrefied, ruptured with pressure), ungerminated, or germinated seeds. Ungerminated seeds were transferred to labeled petri dishes with two sheets of wet filter paper and placed into a growth chamber (CONVIRON G30, Winnepeg, Canada) for 2 weeks set to standard germination conditions (25/15°C, 12/12 h, light/dark). Seeds that did not germinate were subjected to tetrazolium chloride (TZ) for viability testing as in Alexander et al. (2014) (Delouche et al. 1962) and classified as either viable (red staining) or nonviable (nonstaining). Thus, seeds deemed ungerminated but viable when tested with TZ were considered dormant, while unviable seeds were considered dead. For the germinated seeds at the second removal date (early spring), seeds that had germinated in the fall rather than the spring were further classified as fall germinated (seedling and radicle decaying) or early spring germinated (radicle alive). At the spring removal date, we did not observe decaying radicles, and so we could not reliably distinguish fall germinated seeds from seeds that had died and left an empty hull. To unify these sets of data, fall-germinated seeds in the early spring removal were ultimately classified as dead. Empty seeds were easily distinguished from dead seeds at the early spring removal and were subtracted from the total number of seeds in the compartment.

### Statistical analysis

#### Seed burial experiment

First, to detect the effects of removal time and cross type on germinated, ungerminated, and dead seeds (collectively, a categorical variable called seed fate), we used a generalized linear mixed model anova with a multinomial response distribution (PROC GLIMMIX, SAS 2008) to perform maximum-likelihood analyses and obtain least squares estimates of germinated, ungerminated, and dead seed values in the burial study. The late fall removal was excluded from these analyses and analyzed separately because seed fate at this date was influenced mostly by fall germination with very low mortality (ungerminated seed was the inverse of late fall germination). Because the burial study was a split-plot design, for both anova and regressions that included removal, the block by removal interaction was used as an error to test removal. Within a removal date, we compared cross types using a Tukey–Kramer adjustment for multiple comparisons. Due to a significant removal by cross type interaction, we subjected each removal date and seed fate to anova separately using binomial response distributions. We constructed three *a priori* contrasts to further investigate the effect of maternal parent for reciprocal crosses. By examining reciprocal maternal cross type pairs, we could hold % crop alleles constant while more specifically contrasting maternal effects. We contrasted reciprocal cross types with 25% (W × F_1_ vs F_1_ × W), 50% (W × C vs C × W), or 75% (F_1_ × C vs C × F_1_) crop alleles, which differed in their maternal parent (Table1). This approach allowed us to understand how particular fates or removal times were driving the observed interactions. We used regression analyses to elucidate the effects of increasing percentage crop alleles on each seed fate while also assessing effects of removal date, maternal parent, and all their interactions, along with block. This overall model was run along with separate analyses for each removal date.

## Results

### Field seed burial experiment: effects of removal date and cross type on seed fate

**Figure 1:**
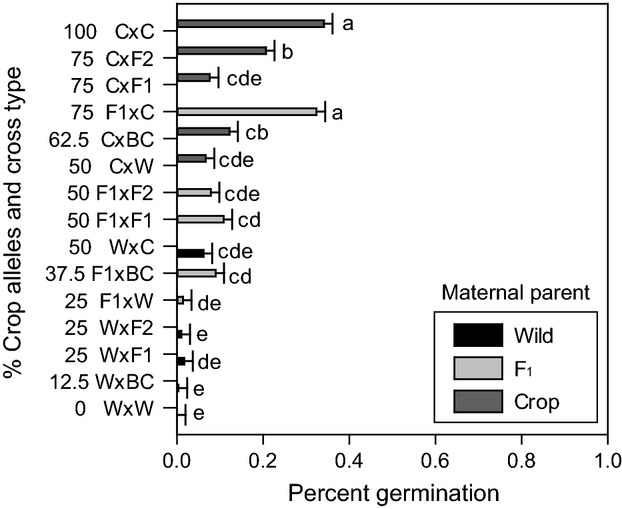
Late-fall germination of sunflower crop–wild cross types. Cross types are organized on the *y*-axis by increasing crop allele percentages as marked. Maternal parent is listed first for each cross type. Germination least squares means (with SE bars) followed by the same letter are not significantly different using a Tukey–Kramer adjustment for multiple comparisons.

**Figure 2:**
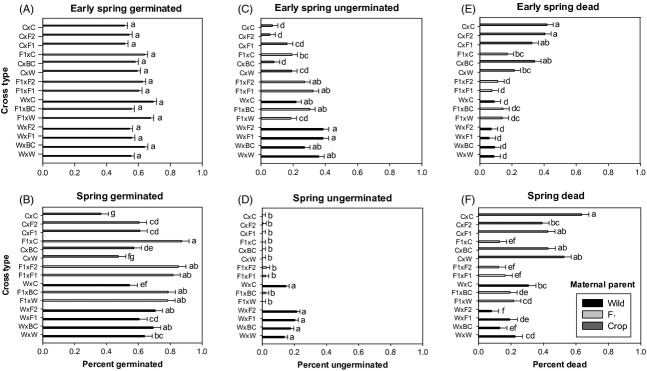
Percent germinated for early spring (A), spring (B), percent ungerminated for early spring (C), spring (D), and percent dead for early spring (E) and spring (F) removal dates. Sunflower crop–wild hybrid cross types are organized by increasing crop allele percentages, with the most crop-like on the top of the *y*-axis and the most wild-like at the bottom of the *y*-axis. Cross type maternal parent is listed first. Least squares means for germinated, ungerminated, and dead seed (with SE bars) followed by the same letter are not significantly different using a Tukey–Kramer adjustment for multiple comparisons.

In the multinomial and binomial analyses of seed fate (germinated, ungerminated, or dead), we found that the interaction between removal date and cross type was consistently significant (Table S1). To better understand this interaction, we investigated each removal date and seed fate individually (Table2) and then used our *a priori* contrasts among reciprocal cross types with identical crop allele percentages to further discern maternal effects. For the late fall removal date, germination was significantly different among cross types (Table2), with four of the five lowest germination rates in wild-produced cross types of low % crop alleles (Fig.1). Only the contrast between reciprocal crosses each with 75% crop alleles was significant, with higher germination in the F_1_-produced cross type than in the crop-produced one (Table2). For the early spring removal date, germination was not significantly different among cross types (Table2), and all cross types had over 50% germination by that time (Fig.2A). Nevertheless, all three *a priori* contrasts of reciprocal crosses were significant. Where present, the F_1_-produced cross type in the reciprocal pair (in 25% and 75% pairs) had greater germination. For the 50% reciprocal cross pairing, the wild-produced cross types germinated more (Table2). Germination differences between cross types were detected at the spring removal (Table2), with the five highest germination levels in F_1_-produced seeds, ranging from 78.2% in F_1_ × W to 87% in F_1_ × C and four of five lowest germination levels in seeds produced on crop maternal parents, ranging from 36.4% in C × C to 60.8% in C × F_2_ (Fig.2B). Only contrasts for the 25% and 75% crop allele pair were significant for germination, with the F_1_-produced seeds still displaying higher germination in both cases.

**Table 2:** Results from anovas using SAS GLIMMIX for effect of block, cross type and three *a priori* contrasts of cross types with differing maternal parents, but identical crop allele percentages for germinated, ungerminated, and dead seeds in sunflower crop–wild hybrids at each of three removal dates, late fall, early spring, and spring

Seed fate	Effect	Late fall				Early spring				Spring			
		df	*F*	*P*	*	df	*F*	*P*	*	df	*F*	*P*	*
Germinated	Block	14, 196	2.41	0.0039	–	14, 190	1.4	0.156	–	14, 192	2.05	0.016	–
	Cross type	14, 196	30.18	<.0001	–	14, 190	1.61	0.08	–	14, 192	9.49	<.0001	–
	25% W × F1 vs F_1_ × W	1, 196	0.01	0.9097	ns	1, 190	3.38	0.0674	W < F_1_	1, 192	6.73	0.0102	W < F_1_
	50% W × C vs C × W	1, 196	0.03	0.8584	ns	1, 190	2.66	0.1044	W > C	1, 192	1.13	0.29	ns
	75% F_1_ × C vs C × F_1_	1, 196	78.37	<.0001	F_1_ > C	1, 190	3.8	0.0526	F_1_ > C	1, 192	15.78	0.0001	F_1_ > C
Ungerminated	Block	1, 196	1.23	0.2572	–	14, 189	1.8	0.042	–	14, 192	2.73	0.0011	–
	Cross type	1, 196	22.93	<0.0001	–	14, 189	9.85	<.0001	–	14, 192	17.06	<.0001	–
	25% W × F_1_ vs F_1_ × W	1, 196	0.26	0.6109	ns	1, 189	15.52	0.0001	W > F_1_	1, 192	47.31	<.0001	W > F_1_
	50% W × C vs C × W	1, 196	2.12	0.1474	ns	1, 189	0.3	0.5818	ns	1, 192	25.73	<.0001	W > C
	75% F_1_ × C vs C × F_1_	1, 196	31.23	<.0001	F_1_ < C	1, 189	0.37	0.5428	ns	1, 192	0	1	ns
Dead	Block	1, 196	2.53	0.0025	–	14, 189	0.97	0.482	–	14, 192	2.42	0.0038	–
	Cross type	1, 196	3.01	0.0003	–	14, 189	10.24	<.0001	–	14, 192	14.08	<.0001	–
	25% W × F_1_ vs F_1_ × W	1, 196	1.05	0.3075	ns	1, 189	2.02	0.1571	W < F_1_	1, 192	0.15	0.699	ns
	50% W × C vs C × W	1, 196	4.09	0.0444	W < C	1, 189	5.08	0.0254	W < C	1, 192	11.68	0.0008	W < C
	75% F_1_ × C vs C × F_1_	1, 196	6.11	0.0143	F_1_ < C	1, 189	7.42	0.007	F_1_ < C	1, 192	22.58	<.0001	F_1_ < C

The column with the *symbol indicates the directionality of the effect, with the more wild-like maternal parent listed first. Maternal parent is listed first for each cross type.

**Figure 3:**
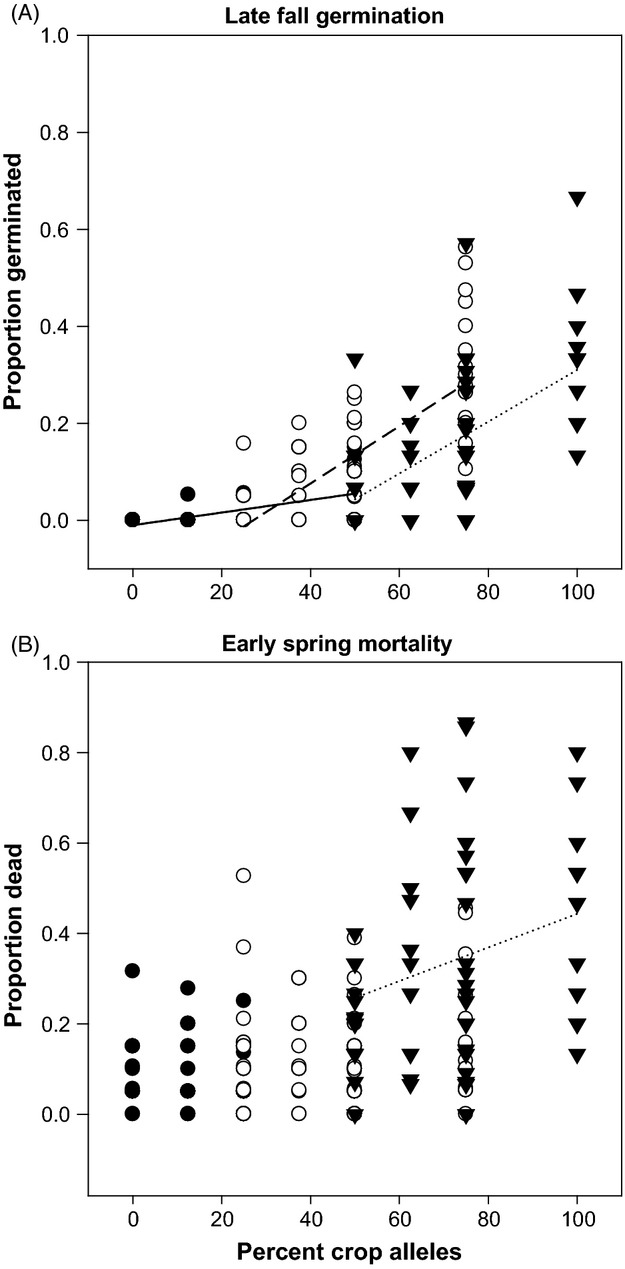
Late-fall germination (A) and early spring mortality (B) as affected by percentages of crop alleles (*x*-axis) and maternal cross type (symbols). Filled circles = wild, unfilled circles = F_1_, and filled triangles = crop. Significant linear regressions (*P *>* *0.05 for effect of crop alleles) are plotted. Solid lines (——) indicate regression lines for wild-produced seeds, dashed lines (– – –) indicate regression lines for F1-produced seeds, and dotted lines (········) indicate regression lines for crop-produced seeds. Equations for all significant regressions are depicted and listed here. (A) Late-fall germination: wild maternal, y = 0.0013x −0.0098 (F_59_ = 42.10, *P* < 0.0001); F_1_ maternal, y = 0.0059x −0.1594 (F_59_ = 86.72, *P* < 0.0001); crop maternal, y = 0.0054x −0.2263 (F_59_ = 47.71, *P* < 0.0001). (B) Early spring mortality: crop maternal, y = 0.0037x +0.0722 (F_59_ = 6.35, *P* = 0.0146).

Ungerminated seed percentages differed among cross types at both spring removals (Tables2 and 3). In the early spring, levels of ungerminated seed in wild- and F_1_-produced seeds was comparable, ranging from 21.7% (W × C) to 38.6% (W × F_2_) in the wild-produced seeds and 18.6% (F_1_ × C) to 32.3% (F_1_ × F_1_) in the F_1_-produced seeds (Fig.2C). By contrast, ungerminated seed in the crop-produced cross types was lower: 5.5% (C × F_2_) to 19% (C × W) (Fig.2C). Only the contrasts for the 25% crop allele pair were significant, with the wild-produced cross type being less likely to germinate than the F_1_-produced cross type (Table2). By later in the spring, none of the crop-produced seeds remained alive but ungerminated; three of the F_1_-produced cross types had very low quantities of ungerminated seed (up to 2.6%) and two had no ungerminated seed (Fig.3D). Of these, negligible levels of truly dormant seeds remained as tested by tetrazolium (Table4). Of the wild-produced seeds, in comparison, 10–20% remained ungerminated and 4–8.8% remained truly dormant depending on the cross type (Table4). Spring contrasts among the 25% and 50% crop allele cross pairs were significant; the wild-produced cross type was less likely to germinate in both cases (Table2).

**Table 3:** Results from a general linear model on sunflower crop–wild hybrid cross types of differing maternal parent and percentage crop alleles that were removed from the soil at three dates: late fall, early spring and spring. SAS GLIMMIX was used to test for effects of block, maternal parent (maternal), percentage of crop alleles (% crop), and their interactions, analyzed by removal date

Seed fate	Effect	Late fall			Early spring			Spring		
		df	*F*	*P*	df	*F*	*P*	df	*F*	*P*
Germinated	Block	14, 28	2	0.0577	14, 28	1.25	0.2983	14, 28	1.91	0.071
	Maternal	2, 28	17.65	<.0001	2, 28	1.2	0.3174	2, 28	0.15	0.8589
	% crop	1, 177	153.14	<.0001	1, 171	0.04	0.8473	1, 173	1.39	0.2402
	% crop*Maternal	2, 177	18.99	<.0001	2, 171	2.83	0.0617	2, 173	3.5	0.0324
Ungerminated	Block	14, 28	1.11	0.388	14, 28	1.48	0.1836	14, 28	1.26	0.2923
	Maternal	2, 28	4.94	0.0146	2, 28	2.1	0.1411	2, 28	16.54	<.0001
	% crop	1, 177	98.95	<.0001	1, 170	6.78	0.0101	1, 173	0.01	0.9404
	% crop*Maternal	2, 177	8.98	0.0002	2, 170	0.88	0.4149	2, 173	0.03	0.9674
Dead	Block	14, 28	0.98	0.4992	14, 28	0.82	0.6443	14, 28	2.12	0.0442
	Maternal	2, 28	6.12	0.0062	2, 28	0.11	0.8961	2, 28	2.17	0.1331
	% crop	1, 177	0.37	0.5428	1, 170	5.21	0.0237	1, 173	1.43	0.2337
	% crop*Maternal	2, 177	2	0.1385	2, 170	3.94	0.0213	2, 173	3.58	0.0301

**Table 4:** Least squares means for the percent dormant fraction of ungerminated seeds at the early spring removal. Seeds were determined to be viable using tetrazolium chloride after incubation in favorable germination conditions

Cross type	TZ dormant	Standard error
W × W	0.071	0.01308
W × BC	0.041	0.0126
W × F1	0.089	0.01361
W × F2	0.054	0.0126
F1 × W	0	0.0126
F1 × BC	0.013	0.0126
W × C	0.075	0.01308
F1 × F1	0.007	0.0126
F1 × F2	0.013	0.0126
C × W	0	0.0126
C × BC	0	0.0126
F1 × C	0	0.0126
C × F1	0	0.0126
C × F2	0	0.0126
C × C	0	0.0126

Late fall mortality was very low overall, but contrasts indicated that wild-produced cross types fared best, followed by F_1_-produced cross types, with crop-produced cross types suffering the highest mortality levels (Table2). At this removal date, differences in seed death were driven by maternal parentage (Table3). The results for mortality were generally symmetrical to those of ungerminated seed at the early spring removal. To accent differences between cross types that, as a whole, were not different for germination, we calculated least squares means for an ungerminated: dead ratio (Fig. S1). Percentages also differed among cross types at both spring removals, but the crop-produced cross types showed the highest levels of mortality at both removal dates (Table2, Fig.2C, D). Mortality increased for nearly all cross types between early spring and spring, although a few cross types experienced no change in this interval (Fig.2C, D). Contrasts among both the 50% and the 75% crop allele cross type pairs indicated that the crop-produced cross type consistently displayed higher mortality across all removal dates when compared with wild- or F_1_-produced cross types (Table2). While only significant for the early spring removal, the F_1_-produced cross type had higher mortality than the wild-produced cross type for the contrast of the 25% crop allele cross type (Table2).

### Regressions of percent crop alleles on seed fates controlling for maternal parent

Initial regressions on each seed fate (germinated, ungerminated, and dead) indicated significant three-way interactions between percentage crop alleles, removal date, and maternal parent (Table S2). To elucidate this interaction, subsequent analyses by removal date (or by removal and maternal parent if interactions between maternal parent and % crop alleles remained significant) were performed. These analyses indicated that late-fall germination increased linearly with % crop alleles for each maternal type (Fig.3), although % crop alleles had a stronger effect on germination for F_1_ and crop maternal parents than for cross types produced on wild maternal plants (Fig.3A). Levels of seeds that remained ungerminated in the late fall was also significantly affected by % crop alleles, but represented the inverse pattern seen for germination for this removal (data not shown). Maladaptive fall germination in wild-produced and F_1_-produced cross types appears to have equalized by the early spring removal, as demonstrated by early spring mortality (Figs2E and 3). By early spring, seed mortality was not affected by percent crop alleles for wild-produced or F_1_-produced cross types, but seeds produced on crop maternal plants had increased mortality with increasing crop alleles (Table3, Fig.3B). Germination patterns for wild-, F_1_-, and crop-produced cross types diverged by spring. Though not significant (Table3), % crop alleles had a negative effect on spring germination of seeds produced on wild and crop maternal plants while having a positive effect on germination of F_1_-produced seeds, which also had higher overall germination (data not shown).

## Discussion

Our work with sunflowers revealed that both percent crop alleles and the identity of the maternal parent (and their interaction) affected levels of germinated, ungerminated, and dead seed. Interactions arose because seeds with a greater percentage of crop alleles tended to be more likely to germinate in the fall and die by the spring, but to differing degrees based on the maternal parent of the cross type. Where detected, differences between reciprocal pairs with equivalent crop allele percentages at each removal revealed that (i) crop-produced cross types displayed the highest mortality, (ii) F_1_-produced cross types showed the highest germination, and (iii) wild-produced cross types had the highest dormancy (Table4). By the end of the field season, only seeds with a wild mother retained measurable dormancy and thus would be able to contribute to a persistent seed bank (Table4). While maternal genetic effects and seed bank dynamics need to be considered when discussing the introgression of crop alleles in a hybrid zone, our results did not indicate any major barriers to introgression due to seed characteristics.

### Maternal genetic and nuclear genetic effects on seed fate

Variation among crop-wild cross types for seed fates have been investigated in a number of ways. Some research has compared arrays of cross types to investigate how hybridization itself can affect germination levels (Landbo and Jørgensen 1997; Hauser and Shim 2007). Generally, this research analyzes cross types as a group and cannot formally discern different forms of genetic effects that could be differentiating the behavior of various hybrid cross types. Others have tried to identify either maternal genetic (Adler et al. 1993) and/or embryo genetic effects (Brunick 2007). For instance, Brunick (2007) found QTLs for embryo dormancy in sunflower, indicating a nuclear genetic role for the maintenance of dormancy. Recent sunflower studies have begun to dissect maternal genetic effects apart from effects imparted by nuclear genetic composition, while also accounting for any interactions (Weiss et al. 2013; Alexander et al. 2014). Our results extend this work in two important ways. First, in contrast to past work, our study utilized both a broad range of 15 sunflower cross types and a realistic field conditions. These features allow us to perform an unusually complete analysis exploring how multiple genetic factors interact to influence seed fate. For example, our regression analyses clarified interactions between the maternal parent and % crop alleles in late fall by showing that while germination increased with % crop alleles, maternal types differed in their maladaptive germination responses (i.e. their slopes in Fig.3). Additionally, by removing seed burial strips at three times over the season, our work is the first to directly quantify premature germination in the fall, compare those data with mortality in the early spring, and hypothesize about the contribution of fall germination to seedling death from a single cohort of seeds.

Past literature has focused on maternal effects, with reports on how they can influence the early life cycle of crop-wild hybrids (via dormancy) in sunflower (Presotto et al. 2011; Weiss et al. 2013) and canola (Adler et al. 1993). Yet maternal parentage can affect other traits, such as seed size, weight, and germination timing (Stanton 1985; Roach and Wulff 1987). Larger seeds (such as those of F_1_- and crop-produced cross types) also produce larger seedlings (Alexander et al. 2014), a trait which in general is associated with greater competitive ability and adult fitness across plant taxa (Stanton 1984, 1985). In our study, we see an apparent timing effect in the F_1_-produced cross types, whose germination increased in the spring relative to the other cross types, and with increasing % crop alleles (Fig.2A, B). More germination later in the spring could push the average emergence date for these cross types later in the season and later emergence may be disadvantageous. Mercer et al. (2011) and Kost (2014) both found evidence that later emergence reduced reproductive potential although emergence timing may be less stringently tied to fitness in F_1_-produced cross types (Kost 2014). Thus, although the F_1_-produced cross types may be at a size advantage for competition, some of them may do poorly due to later emergence. Nevertheless, germination likely overestimates emergence, as germinated seeds may still fail to emerge and the seed burial strips used in this study precluded seed predation.

### No clear barriers to crop allele introgression

Reduced dormancy in crop–wild hybrid seed has been put forth as a barrier to crop allele introgression into wild populations (Stewart 2003). However, the distinction between a barrier to crop allele introgression into wild populations and a resistance or hindrance to crop allele introgression is an important one. A barrier implies a block to introgression, whereas a resistance implies a slowing of the process. As germination during the fall will result in seedling death in most temperate hybrid zones, there would be strong selection against the crop alleles or maternal genetic effects driving this germination timing. Inappropriate germination timing in some but not all hybrid seed would tend to purge maladaptive crop alleles from the population, but retain neutral or adaptive ones. As the late-fall removal illustrates, the F_1_- and crop-produced seeds had higher germination than wild-produced seeds (Fig.1). While it is possible that some seed may have died without germinating, mortality at early spring follows a pattern similar to late-fall germination indicating that this proportion is likely small (Fig.3). By early spring, F_1_-produced seeds have similar mortality to wild-produced seeds, indicating similar overwintering capacity (Fig.2E). Crop-produced cross type seeds displayed levels of mortality at early spring that suggest overwinter germination may constitute a major source of mortality for these cross types (Fig.2E). Nevertheless, many crop-produced cross type seeds had mortality of approximately 60% at the spring removal date (Fig.2F). Thus, higher overwinter mortality in crop-produced cross type seeds represents a resistance to crop allele introgression, rather than a barrier. F_1_-produced cross type seeds overwinter in a fashion similar to wilds (Fig.2E).

The high mortality of seed crop-produced cross types reduced the number of seeds that could germinate. Seed of wild-produced cross types had high dormancy, thus reducing their germination. Either of these two avenues could result in equivalent germination levels at the early spring removal date (Table2), but they can be discerned by the ratio of ungerminated to dead seeds, which displayed considerable and somewhat clinal variation (Fig. S1). A higher ratio of ungerminated to dead seeds indicates an increase in survival capacity of seeds, which reduces the risk of total reproductive failure by spreading germination over multiple seasons via dormancy. Notably, hybrid cross types with the highest and lowest ungerminated : dead ratio were W × F_1_ (25%) and C × F_2_ (75%), respectively (Fig. S1). Similar to our results, but with a related metric, a low dormancy: mortality ratio was calculated for canola crop–wild hybrids, while a high dormancy: mortality ratio was found in its wild relative (Adler et al. 1993). High overwinter survival and subsequent early spring germination (∽50%) from all three maternal types indicate that seeds from all cross types may have the potential to emerge the following spring, with wild-produced cross types able to enter the seed bank portion of the life cycle. This result confirms a smaller seed burial study by Alexander et al. (2014). Studies have shown that crop–wild hybrid sunflower seedlings can be larger than wild seedlings and, depending on the environment, nearly match wilds in fitness (Mercer et al. 2007, Mercer et al. 2014). Thus, following a hybridization event where wild, F_1_, and crop plants each produce crop–wild hybrid seeds, the substantial number of surviving seedlings has the potential to cross and backcross with each other. We find no evidence of seed dormancy presenting a serious hurdle for introgression of crop alleles into wild population in future generations, although some hybrid generations may better facilitate introgression through the seed bank.

### Introgression facilitated by a persistent seed bank

While overwinter dormancy is an important determinant of survival, dormancy over multiple seasons has the greatest potential to extend the effects of an isolated hybridization event. True dormancy was highest at the spring removal date in wild-produced seeds, minimal in F_1_-produced seeds, and absent in crop-produced seeds (Table4). Although sunflower seed banks influence population dynamics most in the first few years after their establishment, sunflower longevity in the seed bank can be lengthy (Alexander and Schrag 2003). For example, in agricultural environments where plowing may bury seeds, up to 15% of seeds dispersed in a given year may be able to germinate 10 years later (Teo-Sherrill 1996). In a long-term burial study, Burnside et al. (1996) found that 3% of seeds buried at 20 cm were able to germinate after 17 years. While we cannot predict long-term dormancy from our data (additional germination and mortality after the final removal date was likely), nevertheless, only seeds produced on wild maternal parents are likely to contribute to a persistent seed bank. Thus, only wild-produced hybrid cross types will be capable of germinating beyond the first spring following hybridization, which could facilitate introgression. The potential for dormancy to extend into future years in wild-produced seeds that we have observed here suggests that wild seed traits may facilitate crop allele introgression by conveying wild-like dormancy to hybrids. However, seedlings produced from crop parents would inherit the crop cytoplasm, organelles, and chloroplasts, which may themselves provide fitness advantages to future generations after recombination purges low-fitness traits (Allainguillaume et al. 2009; Mercer 2009). Introgression of crop alleles has already occurred in sunflower hybrid zones and will likely continue (Lai et al. 2012).

Our work with diverse hybrid cross types under field conditions provides the clearest idea to date of the ways maternal and nuclear genetic effects can influence the seed stage in a crop–wild hybrid zone. Ultimately, levels of introgression for any given crop allele will depend on its effects on the lifetime fitness, including the seed stage, in each generation through which it passes. Field studies using this kind of diversity of cross types, but allowing for emergence after overwintering and measures of fitness will further clarify introgression dynamics.

## Data archiving statement

Data available from the Dryad Digital Repository: http://doi.org/10.5061/dryad.d06j2

## Supporting Information

**Figure S1.** Early spring ungerminated:dead ratio for each cross type.

**Table S1.** Results from Multinomial seed fate (germinated, ungerminated and dead) anovas using SAS GLIMMIX across two removal dates, early spring and spring, compared with anovas for individual seed fates of germination, dormancy and mortality.

**Table S2.** Results from anovas of sunflower crop-wild hybrid seeds of differing maternal parent and percentage crop alleles removed from soil at three removal dates, late fall, early spring and spring.
