# Supplementary material for: Seed fates in crop–wild hybrid sunflower: crop allele and maternal effects
Source: Evol Appl. 2014 Dec 5;8(2):121–32. doi: 10.1111/eva.12236 (PMC4319861; doi:10.1111/eva.12236)
Supplement: Supplementary file 1 [file eva0008-0121-sd1.docx]

**Supplementary Figure 1.** Early spring ungerminated:dead ratio for each cross type. ungerminated:dead ratios for each cross type were produced using SAS GLIMMIX. Least squares means (with s.e. bars) followed by the same letter are not significantly different using a Tukey-Kramer adjustment for multiple comparisons.
