# Supplementary material for: Seed fates in crop–wild hybrid sunflower: crop allele and maternal effects
Source: Evol Appl. 2014 Dec 5;8(2):121–32. doi: 10.1111/eva.12236 (PMC4319861; doi:10.1111/eva.12236)
Supplement: Supplementary file 2 [file eva0008-0121-sd2.docx]

**Supplementary Table 1.** Results from Multinomial seed fate (germinated, ungerminated and dead) ANOVAs using SAS GLIMMIX across two removal dates, early spring and spring, compared with ANOVAs for individual seed fates of germination, dormancy and mortality. Block, removal, cross type and removal by cross (cross type) interactions were determined by using removal as the main plot treatment and cross type as the subplot treatment.

|  | Multinomial | | | Germinated | | | Ungerminated | | | Dead | | |
| --- | --- | --- | --- | --- | --- | --- | --- | --- | --- | --- | --- | --- |
| Effect | DF | F | P | DF | F | P | DF | F | P | DF | F | P |
| Block | 14, 14 | 1.33 | 0.3016 | 14,196 | 1.02 | 0.432 | 14,196 | 1.59 | 0.084 | 14,196 | 1.34 | 0.189 |
| Removal | 1, 14 | 0.9 | 0.3585 | 1,200 | 18.83 | <.0001 | 1,199 | 244.2 | <.0001 | 1,199 | 36.14 | <.0001 |
| Cross type | 14, 7290 | 32.12 | <.0001 | 14,196 | 6.98 | <.0001 | 14,196 | 18.72 | <.0001 | 14,196 | 21.2 | <.0001 |
| Removal*Cross | 14, 7290 | 10.75 | <.0001 | 14,200 | 4.06 | <.0001 | 14,199 | 3.68 | <.0001 | 14,199 | 2.55 | 0.0022 |
