# Supplementary material for: Seed fates in crop–wild hybrid sunflower: crop allele and maternal effects
Source: Evol Appl. 2014 Dec 5;8(2):121–32. doi: 10.1111/eva.12236 (PMC4319861; doi:10.1111/eva.12236)
Supplement: Supplementary file 3 [file eva0008-0121-sd3.docx]

**Supplementary Table 2.** Results from ANOVAs of sunflower crop-wild hybrid seeds of differing maternal parent and percentage crop alleles removed from soild at three removal dates, late fall, early spring and spring. SAS GLIMMIX was used to test for effects of removal, maternal parent, percentage crop alleles (% crop), and their interactions.

|  | Germinated | | | Ungerminated | | | Dead | | |
| --- | --- | --- | --- | --- | --- | --- | --- | --- | --- |
| Effect | DF | F | P | DF | F | P | DF | F | P |
| Block | 14, 28 | 0.6 | 0.8392 | 14, 28 | 1.13 | 0.3756 | 14, 28 | 0.86 | 0.61 |
| Removal | 2, 28 | 172.04 | <.0001 | 2, 28 | 462.51 | <.0001 | 2, 28 | 9.17 | 0.0009 |
| Maternal | 2, 605 | 0.2 | 0.8218 | 2, 604 | 2.57 | 0.0771 | 2, 604 | 2.95 | 0.0533 |
| % crop | 1, 605 | 10.32 | 0.0014 | 1, 604 | 50.82 | <.0001 | 1, 604 | 4.71 | 0.0304 |
| Removal*Maternal | 4, 605 | 3.38 | 0.0095 | 4, 604 | 6.88 | <.0001 | 4, 604 | 0.88 | 0.4769 |
| % crop*Maternal | 2, 605 | 4.23 | 0.0149 | 2, 604 | 1.58 | 0.2073 | 2, 604 | 4.77 | 0.0088 |
| % crop*Removal | 2, 605 | 18.87 | <.0001 | 2, 604 | 21.7 | <.0001 | 2, 604 | 1.81 | 0.1645 |
| % crop*Removal*Maternal | 4, 605 | 5.01 | 0.0006 | 4, 604 | 3.71 | 0.0054 | 4, 604 | 2.94 | 0.0201 |
